# Rapid identification of respiratory bacterial pathogens from bronchoalveolar lavage fluid in cattle by MALDI-TOF MS

**DOI:** 10.1038/s41598-019-54599-9

**Published:** 2019-12-05

**Authors:** Laura Van Driessche, Jade Bokma, Piet Deprez, Freddy Haesebrouck, Filip Boyen, Bart Pardon

**Affiliations:** 1 Department of Large Animal Internal Medicine, Faculty of Veterinary Medicine, Ghent University, Salisburylaan 133, 9820 Merelbeke, Belgium; 2 Department of Pathology, Bacteriology and Avian Diseases, Faculty of Veterinary Medicine, Ghent University, Salisburylaan 133, 9820 Merelbeke, Belgium

**Keywords:** Infectious-disease diagnostics, Clinical microbiology, Respiratory tract diseases

## Abstract

Respiratory tract infections are a major health problem and indication for antimicrobial use in cattle and in humans. Currently, most antimicrobial treatments are initiated without microbiological results, holding the risk of inappropriate first intention treatment. The main reason for this empirical treatment is the long turnaround time between sampling and availability of identification and susceptibility results. Therefore the objective of the present study was to develop a rapid identification procedure for pathogenic respiratory bacteria in bronchoalveolar lavage fluid (BALf) samples from cattle by MALDI-TOF MS, omitting the cultivation step on agar plates to reduce the turnaround time between sampling and identification of pathogens. The effects of two different liquid growth media and various concentrations of bacitracin were determined to allow optimal growth of *Pasteurellaceae* and minimise contamination. The best procedure was validated on 100 clinical BALf samples from cattle with conventional bacterial culture as reference test. A correct identification was obtained in 73% of the samples, with 59.1% sensitivity (Se) (47.2–71.0%) and 100% specificity (Sp) (100–100%) after only 6 hours of incubation. For pure and dominant culture samples, the procedure was able to correctly identify 79.2% of the pathogens, with a sensitivity (Se) of 60.5% (45.0–76.1%) and specificity (Sp) of 100% (100–100%). In mixed culture samples, containing ≥2 clinically relevant pathogens, one pathogen could be correctly identified in 57% of the samples with 57.1% Se (38.8–75.5%) and 100% Sp (100–100%). In conclusion, MALDI-TOF MS is a promising tool for rapid pathogen identification in BALf. This new technique drastically reduces turnaround time and may be a valuable decision support tool to rationalize antimicrobial use.

## Introduction

Respiratory tract infections are a leading health issue worldwide, both in humans and animals^1–3^. Also in cattle respiratory tract infections have a major impact on farm economics and animal welfare^4^. Their role as main indication for antimicrobial use in this species is especially important from a One Health perspective^5^. In several food animal industries, antimicrobial resistance is widespread in commensal, pathogenic, and zoonotic bacteria^6,7^. To rationalize antimicrobial use for treatment of respiratory tract infections, rapid availability of microbiological results and antimicrobial susceptibility data are equally important in animals and humans. Respiratory tract samples are recommended to guide antimicrobial use^8^. Different techniques are available, of which bronchoscopic bronchoalveolar lavage is among the most frequently used one^9^. In cattle, a non-endoscopic bronchoalveolar lavage (nBAL), using custom-made low cost catheters, has been developed to meet the demands of veterinary farming practices for a reliable, simple, inexpensive and safe diagnostic technique^10^.

Most initiated antimicrobial therapies for respiratory tract infections are empirical, meaning that antimicrobials, based on collective experience, are provided before microbiological results are available. This is due to the long turnaround time between sampling and availability of culture and susceptibility testing results, which takes at least 48 hours. A reduction in turnaround time is crucial to avoid inappropriate antimicrobial treatment and has been associated with faster adjustment of this treatment and a shorter intensive care unit stay of patients^11^.

In recent years, Matrix-Assisted Laser Desorption/Ionization-Time of Flight Mass Spectrometry (MALDI-TOF MS) has revolutionized microbiology routine practice by reducing the turnaround time at different levels. First, identification of clinically relevant bacteria after standard culture on agar plates is performed much faster^12,13^. Second, with the MALDI Biotyper antibiotic susceptibility test rapid assay (MBT-ASTRA) method, which compares growth of a bacterium with and without an antimicrobial in order to detect resistance, a susceptibility test result can be reached in only few hours^14,15^. To further reduce turnaround time, and achieve the ultimate goal of availability of microbiological identification within one working day, fast identification of the organism from the sample is essential. Protocols for rapid detection of bacteria in clinical samples by MALDI-TOF MS, skipping cultivation on agar plates, are currently available for blood^16^, urine^17^ and other body liquids like peritoneal, synovial and cerebrospinal fluid^18^. To date, no such technique has been developed for broncho-alveolar lavage fluid (BALf) in humans or animals, likely because of the presumed more polymicrobial nature (more contamination expected) of these samples and higher prevalence of mixed infections^10^. Given the important role respiratory tract infections play in worldwide antimicrobial use, the objective of the present study was to develop and evaluate a MALDI-TOF MS technique for rapid identification of respiratory pathogenic bacteria in BALf samples from cattle.

## Results

### Protocol development and optimization

Detection of bacteria depends on the presence of contaminants. Therefore, development consisted of adding an appropriate antimicrobial to minimise bacterial contamination. Since bacitracin has been described to minimise contaminating organisms in respiratory samples in cattle^19^, this antimicrobial was also used in this study. The optimal concentration of bacitracin, both minimising bacterial contamination of the sample and allowing *Pasteurellaceae* growth, was determined. After 6 hours of incubation, no difference in growth (expressed by CFU/mL) was seen between the various concentrations of bacitracin for *Pasteurella multocida*, *Mannheimia haemolytica* and *Histophilus somni*. In comparison with the starting concentration of 1 × 10^4^ CFU/mL, growth increased with 2–4 logs after 6 hours of incubation (mean log concentration: 7.83; standard deviation: 0.22). A concentration of 32 µg/mL was used for further validation.

**Figure 1:**
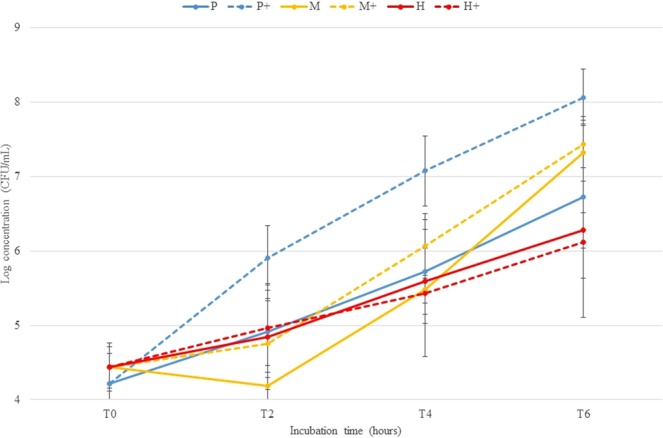
Comparison of BHIB (P, M, H) and BHIB supplemented with 10% fetal bovine serum and 0.5% yeast extract (P+, M+, H+) for *P. multocida* (P), *M. haemolytica* (M) and *H. somni* (H) at different incubation times (0-2-4-6 hours) to optimise bacterial growth (CFU/mL) allowing rapid detection with MALDI-TOF MS. Presented values are the mean of the two strains and the repetitions made including standard deviations.

Since the concentration of pathogens in bovine nBAL samples is generally considered low (average of 1 × 10^4^ CFU/mL^20^), and the MALDI-TOF MS technique requires high bacterial counts to provide reliable results^21^ (a minimum of 1 × 10^7^–1 × 10^8^ CFU/mL for *Pasteurellaceae*, data not shown), an incubation step in growth medium is mandatory. Therefore, 2 different growth media were examined. For *P. multocida*, better growth was observed in the supplemented Brain heart infusion broth (BHIB) compared to BHIB (Fig. 1). For *M. haemolytica* and *H. somni* no difference in growth was observed between BHIB and supplemented BHIB. Considering the better results for *P. multocida*, the supplemented BHIB was selected for the validation study. In addition, an incubation period of 6 hours was selected for the validation study, given the fact that *P. multocida* and *M. haemolytica* concentrations did not reach the detection limit of MALDI-TOF MS (i.e. 1 × 10^7^–1 × 10^8^ CFU/mL) after 4 hours of incubation. Longer incubation periods might not have a clear added value over standard cultivation on plate, since this will probably not lead to identification on the same day of sample inoculation.

### Validation on 100 clinical BALf samples

Out of the 100 BALf samples collected, 45 different bacterial species were isolated. Seven were considered as clinically relevant pathogens in cattle (*M. haemolytica*, *P. multocida*, *Bibersteinia trehalosi*, *Moraxella ovis*, *Mannheimia varigena*, *H. somni* and *Gallibacterium anatis*). A pure culture, meaning the presence of only 1 bacterial pathogen with a concentration of ≥2 × 10^1^ CFU/mL BALf, was obtained in 14% of the samples. A dominant culture, where 1 clinically relevant pathogen was abundantly present, amongst contaminants, which could be easily subjected to subculture for further identification, was seen in 24% of the samples. The majority of the samples obtained was defined as a mixed culture (28%), meaning the presence of ≥2 clinically relevant pathogens, possibly amongst contaminants, which could be easily subjected to subculture for further identification. In 18% of the samples, the culture was polymicrobial, meaning the growth of mainly contaminants and no dominant presence of clinically relevant bacteria and in 16% of the samples the culture result was negative (≤1 × 10^1^ CFU/mL BALf). Correct identification rates of the clinically relevant pathogens by the rapid MALDI-TOF MS procedure are listed in Table 1. Detailed information on the identification results of the conventional culture and the rapid MALDI-TOF MS method can be found in the Supplementary Data, Table 1. Considering pure culture samples only, correct rapid identification with MALDI-TOF MS was possible in 71% of the samples, with a Se of 71.4% (47.8–91.1%) and Sp of 100% (100–100%). When a dominant culture was obtained, correct identification occurred in 58% of the samples, with a Se of 54.2% (34.2–74.1%) and Sp of 100% (100–100%). In pure and dominant cultures, with consideration of the negative culture results correctly identified, the proportion of observed agreement was 79.2%, with a Se of 60.5% (45.0–76.1%) and a Sp of 100% (100–100%) (Table 2).

**Table 1:** Identification of clinically relevant pathogens from BALf samples by conventional culture and rapid detection with MALDI-TOF MS.

Microorganism	Total number isolated in conventional bacteriological culture	Correct MALDI ID compared to all cultures	Correct MALDI ID for pure and dominant cultures	Correct MALDI ID for mixed cultures
		Number (Percentage)	Number (percentage)	Number (Percentage)
*Mannheimia haemolytica*	27	14/27 (51.9%)	7/9 (77%)	7/18 (38.8%)
*Pasteurella multocida*	18	11/18 (61.1%)	7/7 (100%)	4/11 (36.4%)
*Bibersteinia trehalosi*	9	7/9 (77.8%)	3/3 (100%)	4/6 (66.7%)
*Moraxella ovis*	22	0/22 (0%)	0/8 (0%)	0/14 (0%)
*Mannheimia varigena*	8	1/8 (12.5%)	1/3 (33.3%)	0/5 (0%)
*Histophilus somni*	12	1/12 (8.3%)	0/3 (0%)	1/9 (11.1%)
*Gallibacterium anatis*	2	2/2 (100%)	2/2 (100%)	ND

ND: not detected.

**Table 2:** 2 × 2 contingency table for rapid MALDI-TOF MS identification as index test compared to bacterial culture as reference test for identification of respiratory pathogens in pure and dominant BALf culture samples of cattle.

Pure and dominant cultures only	Bacterial culture +	Bacterial culture −^a^	Total
Rapid MALDI-TOF MS+	23	0	23
Rapid MALDI-TOF MS−	15	34	49
Total	38	34	72

^a^All polymicrobial and negative cultures. When MALDI-TOF MS identified a contaminant, the result was considered rapid MALDI-TOF MS−.

**Figure 2:**
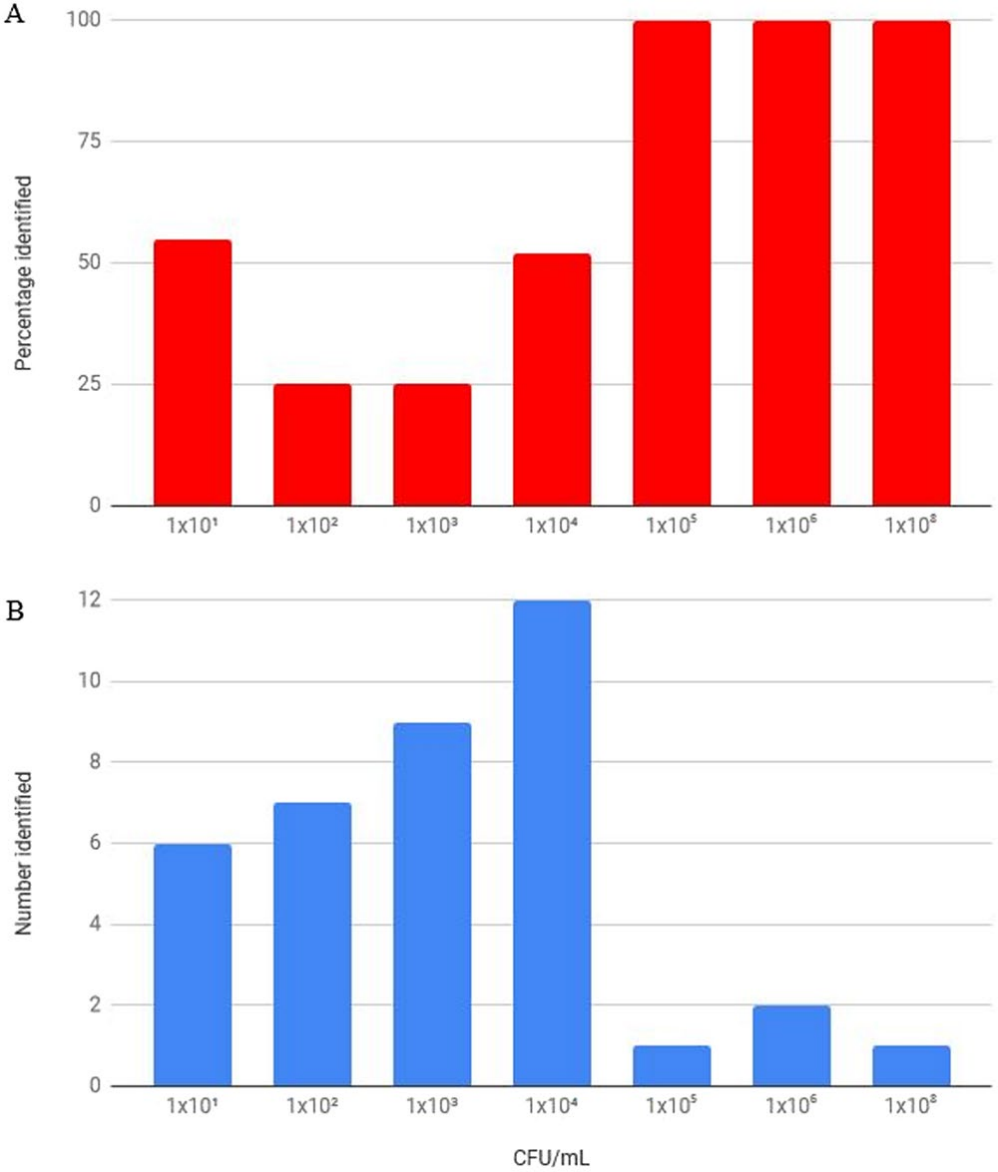
Effect of initial pathogen concentration (CFU/mL) in BALf on the percentage of correct identification rate (**A**) and number of correct identification rate (**B**) with the rapid MALDI-TOF MS procedure.

Twenty-eight percent of the clinical samples contained a mixed culture with 2, 3 or 4 different clinically important pathogens in 22, 5 and 1 mixed culture sample, respectively. In mixed cultures, the correct identification rate per clinically relevant pathogen was considerably lower, since MALDI-TOF MS only identified one pathogen (Table 1). Correct identification of one clinically important pathogen occurred in 57% of the cases, and Se and Sp were 57.1% (38.8–75.5%) and 100% (100–100%), respectively. All polymicrobial and negative samples were correctly classified. *M. ovis*, *H. somni* and *M. varigena* could rarely to not be identified with the rapid MALDI-TOF MS method (Table 1). Taking all clinical samples into account, the proportion of observed agreement between culture and the direct MALDI-TOF MS method was 73%, with a Se of 59.1% (47.2–71%) and a Sp of 100% (100–100%) (Table 3). In Fig. 2, identification percentages stratified on pathogen concentration in BALf are presented. For a bacterial pathogen concentration of 1 × 10^5^ CFU/mL or more in the original sample, correct identification occurred in all cases. For mixed cultures, a positive association was found between the concentration of the pathogen and the rate of correct identification.

**Table 3:** 2 × 2 contingency table for direct MALDI-TOF MS identification as index test compared to bacterial culture as reference test for identification of respiratory pathogens in 100 clinical BALf samples of cattle.

	Bacterial culture +^a^	Bacterial culture −^b^	Total
Rapid MALDI-TOF MS+	39	0	39
Rapid MALDI-TOF MS−	27	34	61
Total	66	34	100

^a^All samples with isolation of a clinically relevant pathogen, meaning pure culture, dominant culture and mixed culture. For mixed cultures, when one pathogen was correctly identified by MALDI-TOF MS, the result was considered direct MALDI-TOF MS+. ^b^All polymicrobial and negative cultures. When MALDI-TOF MS identified a contaminant, the result was considered direct MALDI-TOF MS−.

## Discussion

This study showed that next to blood and urine samples^22–24^, rapid identification of bacterial pathogens by MALDI-TOF MS, skipping the cultivation step on agar, is possible for BALf samples. Taking all samples into account, 73% was correctly identified after 6 hours of incubation, which would be a clinically desirable turnaround time for treatment initiation, since this could result in identification on the same day the sample was inoculated. False positive results did not occur, and 3/5 infections were correctly identified. These results were obtained using a cutoff value of ≥1.7. Although a (log) score value between 1.7 and 2.0 should be interpreted as species identification with low confidence, this cutoff value or even lower is commonly used for identification procedures of positive blood cultures by MALDI-TOF MS^25,26^ and was therefore also applied in the present study.

A limitation of the study was the relatively small sample size, which reduced the number of isolates available for some target pathogens. Whereas Se was above 75% to even 100% for common pathogens like *M. haemolytica* and *P. multocida*, it was below 10% for other bacteria, such as *M. ovis* and *H. somni*, resulting in a lower Se of the technique when considering all samples with pure or dominant cultures. A likely reason why identification rates for *H. somni* and *M. ovis* were poor, is the slow growth rate of these bacteria. Indeed, *H. somni* was shown to reach a concentration of 1 × 10^6^ CFU/mL after 6 hours of incubation, starting from a 1 × 10^4^ CFU/mL inoculum, thereby not reaching the 1 × 10^7^–1 × 10^8^ CFU/mL detection limit (Fig. 1). Therefore, successful detection of *H. somni* by the rapid method depended on the initial concentration in BALf and the presence of other clinically relevant pathogens in the sample. Another reason why identification rates for *H. somni*, *M. varigena* and *M. ovis* were low could be due to the small number of spectra present in the Biotyper database, namely 1 entry for *M. ovis* and *M. varigena* and 2 entries for *H. somni*. Therefore possible solutions for increasing the identification rate of these pathogens could be on the one hand increasing the number of entries of each pathogen involved in the database, and on the other hand expanding the incubation time and perhaps the use of other optimised growth media. Another solution to increase identification rates could be a short incubation on agar plates, as previously described for positive blood culture bottles^27^. However, *Pasteurellaceae* are considered fastidiously growing pathogens, in contrast with the fast growing pathogens like *Enterobacteriaceae*, enterococci and staphylococci that were mainly observed in the previous study^27^. Indeed, preliminary tests in our lab suggest that a similar procedure for *Pasteurellaceae* would probably take too long to have an added value over an overnight culture, even though these observations need confirmation. Additionally, differentiating between multiple pathogens among these young subcultures would be difficult, making this method challenging in samples with contaminants or mixed cultures.

Surprisingly, in six samples a correct MALDI-TOF MS identification was obtained from a starting concentration of 1 × 10^1^ CFU/mL for *M. haemolytica*, *M. varigena*, *P. multocida* and *B. trehalosi*. Given the expected growth rate of the involved species, the most likely explanation for this observation would be that the quantification of 1 × 10^1^ CFU/mL did not represent the true concentration in the BALf, for example due to clumping of bacteria in the sample.

An important limitation of the rapid MALDI-TOF technique is that diagnostic accuracy was substantially lower in mixed culture samples. Similar results were previously observed for blood and urine samples^23,24,28^, but its impact on Se and Sp was not shown in these studies. Even though this can vary between studies, often a single bacterial pathogen is present in the lower respiratory tract of cattle. Mixed infections can occur, but do not make up the majority, which is demonstrated in a study were nBAL samples were taken from 144 preweaned calves with respiratory disease, where 16 samples (=11.1%) obtained a mixed culture^10^. Since mixed cultures can occur in BALf, it is advised to run the classic bacterial culture in parallel with the rapid detection method. However, as correct identification of one species was still possible in 57% of the samples, depending on the species and initial concentration of the respiratory pathogen, the technique can have an added value for mixed cultures as well. Additionally, new techniques are currently being developed addressing the problem of identifying multiple pathogens within one sample^29^, leading to promising results of higher diagnostic accuracy by MALDI-TOF MS in the future, also for mixed samples.

Although the current technique only provides identification of respiratory pathogens, it might provide a basis for rapid antimicrobial susceptibility testing such as the MBT-ASTRA method, especially for pure cultures. Because enriched cultures containing a pure culture will only be detected as such by MALDI-TOF MS when a sufficiently high concentration is obtained (10^7^–10^8^ CFU/mL), the MBT-ASTRA method might be performed subsequently on the same culture, since similar concentrations have been described as inoculum for the MBT-ASTRA method for *Pasteurella multocida*^15^.

In contrast with human medicine, a different classification of samples was applied in the current study. In human medicine, polymicrobial samples are defined as the presence of >1 pathogen in a sample. Since blood cultures and urine samples are normally sterile, and considering the aseptical method of taking these samples, contamination is only rarely encountered. Therefore, when infection is present, mostly 1 pathogen is derived from the sample. In respiratory tract samples of cattle, the presence of >1 clinically relevant pathogen can occur, and is therefore defined as a mixed culture. A polymicrobial culture is seen as the presence of different micro-organisms who are not clinically relevant. Also, a dominant culture can be present, meaning that a clinically relevant pathogen can be isolated, although still some bacterial contaminants can be present in the sample, due to the sampling procedure.

With the nBAL field sampling technique for cattle, approximately 20% of the samples return a polymicrobial test result^10^. However, the nBAL technique was deliberately chosen in the present study since it has been shown to give more pure culture and less polymicrobial culture results compared to other techniques frequently used in cattle such as the deep nasopharyngeal swab^10^. Possible solutions for reducing polymicrobial samples during sampling could be a more hygienic handling or the implementation of endoscopic-guided bronchoalveolar lavages, although the latter method can also not exclude contamination^30^. Additionally, bacitracin was added to the samples during incubation in order to limit contamination, which indeed reduced the presence of most Gram-positive contaminants present in the initial samples. After 6 hours of incubation of the samples, *Escherichia coli* and *Bacillus spp*. were the most common contaminants present (data not shown). Finding a method to minimise the growth of latter bacteria without influencing the growth of *Pasteurellaceae*, would indeed increase the diagnostic accuracy of this new rapid technique. This seems, however, very challenging, since *Pasteurellaceae* are commonly more sensitive to various antimicrobial substances than *Enterobacteriaceae*. Nevertheless, the rapid detection protocol by MALDI-TOF MS obtained no false positive result for all polymicrobial samples. Likely, in humans, the issue of sample contamination would be less prominent than in the currently used farm setting.

This study shows that MALDI-TOF MS considerably improves turnaround time, from 24–48 hours to 6.5 hours in total, bringing identification of causal bacteria of lower respiratory tract infections into a clinically desirable timeframe. Furthermore, with respect to the implementation in the laboratory, the most time-consuming steps (6 hours of incubation, 15 minutes centrifugation) require no hands-on time, making this technique easily applicable in current clinical laboratory workflows. In total 3/5 infections were correctly identified by the rapid MALDI-TOF MS technique. In order to support the decision making process of initiating antimicrobial treatment, the classic bacterial culture can be run in parallel with the rapid detection method. However, no false positive results did occur, leading to a specificity of 100%, and antimicrobial treatment should be initiated when a bacterial compound is present in BALf samples. When an animal with a positive result by the rapid MALDI-TOF MS method is treated the same day, this will probably result in less animal suffering and a more effective treatment. Further research including clinical data on the positive outcome of this reduced turnaround time in veterinary medicine, i.e. reduced time of disease and reduced inappropriate empirical treatment in combination with an increased therapy success, is encouraged. Additionally, the currently described protocol can be used in future studies aiming at different bacteria and host species, including humans.

In conclusion, MALDI-TOF MS is a promising technique for rapid detection of respiratory pathogens in BALf, as demonstrated in cattle. This offers the possibility to practitioners and clinicians to better target their initial antimicrobial treatment.

## Methods

### Protocol development and optimization

Considering the average concentration of target pathogens in bovine BALf samples varies around 1 × 10^4^ CFU/mL^20^, whereas MALDI-TOF MS requires a high concentration for reliable identification of pathogens^21^ (minimum 1 × 10^7^–1 × 10^8^ CFU/mL, data not shown), and considering a doubling time of approximately 30 minutes, a selective enrichment step of maximum 6 hours was deemed necessary to obtain reliable MALDI-TOF identification and still practically feasible for a single-day protocol.

#### Selection of the optimal bacitracin concentration to minimize contaminant growth

Bacitracin was chosen as selective agent, given its previous successful use to minimise contamination for respiratory samples from cattle on agar plates^19^. Isolates used in this study were retrieved from a database consisting of clinical isolates of cattle retrieved by non-endoscopic bronchoalveolar lavage. Isolates were stored at −80 °C. Starting from a fresh overnight culture, *P. multocida* (Pm 187), *M. haemolytica* (Mh 171) and *H. somni* (Hs 12) were each inoculated in 10 mL of Brain heart infusion broth (BHIB, Difco, BD Diagnostic Systems, Sparks, Md.) supplemented with 0, 8, 16 and 32 µg/mL bacitracin at a final concentration of 1 × 10^4^ CFU/mL. Immediately after inoculation and after 6 hours of incubation at a temperature of 35 °C+/−2 °C and an atmosphere enriched with 5% CO_2_, 1 mL sample of each tube was transferred to an eppendorf tube, and ten-fold dilutions were made of each sample for quantitative analysis as previously described^31^. This experiment was performed 2 times independently.

#### Selection of the optimal growth medium for Pasteurellaceae

Two different growth media and the incubation time were examined. BHIB and BHIB supplemented with 10% fetal bovine serum (FBS, Hyclone^TM^ Fetal Bovine Serum, GE Healthcare life sciences, UK, Ltd.) and 0.5% yeast extract (YE, Bacto^TM^ Yeast Extract Technical, BD Diagnostic Systems, Sparks, Md.) were used as growth media. Two strains of *P. multocida* (Pm 180, Pm 182), *M. haemolytica* (Mh 171, Mh 178) and *H. somni* (Hs 12, Hs 15) were inoculated in both media at a starting concentration of 1 × 10^4^ CFU/mL. No antibiotics were added. All tubes were placed in a shaking incubator for 6 hours (35 °C+/−2 °C, 5% CO_2_). One mL of each tube was transferred to eppendorf tubes 0, 2, 4 and 6 hours after inoculation for quantitative analysis as previously described^31^. This experiment was repeated twice.

### Validation on 100 clinical BALf samples

#### Sample collection

Samples originated from cattle (100 different animals, 10 days to 4 years old) from 10 farms with a history of respiratory tract infections. Animals that were treated during the 14-day period prior to sampling were excluded from the study. One hundred nBAL samples were taken from cattle as previously described^32^. Briefly, the nostril was disinfected with 90% alcohol, and a home-made catheter was inserted medioventrally in the nasal cavity. The catheter was further advanced through larynx and trachea into the bronchi until the wedge position was reached. A volume of approximately 0.6 mL/kg body weight of sterile 0.9% NaCl was injected into the lungs and immediately aspirated. Samples were transported at ambient temperature and processed within 14 hours after sampling. The sampling method was approved by the ethical committee of the Faculty of Veterinary Medicine, Ghent University (EC 2019-1). All methods were performed in accordance with the relevant guidelines and regulations.

#### Sample processing

All samples were simultaneously analysed using conventional bacteriology procedures on the one hand and the new MALDI-TOF MS rapid detection protocol, as described below, on the other hand.

Conventional bacterial culture:All nBAL samples were vortexed for 30 seconds and 1 mL was transferred to an Eppendorf tube. Ten-fold dilutions were made of each sample for quantitative analysis as previously described^31^. From each dilution, 100 µl was inoculated on Columbia agar supplemented with 5% sheep blood (blood agar; Oxoïd, Hampshire, UK) and incubated for 24–48 hours at 35 °C +/−2 °C in a 5% CO_2_ atmosphere. All macroscopically different colonies were counted and identified with MALDI-TOF MS by direct transfer of the colony on the target plate with a toothpick^33^. Culture results were classified as (1) negative result (≤1 × 10^1^ CFU/mL BALf), (2) pure culture (presence of only 1 bacterial pathogen with a concentration ≥2 × 10^1^ CFU/mL BALf), (3) polymicrobial result (growth of mainly contaminants and no dominant presence of clinically relevant bacteria) (4) dominant culture (abundant presence of 1 clinically relevant pathogen, amongst contaminants, which could be easily subjected to subculture for further identification) (5) Mixed cultures (two or more clinically relevant pathogens, possibly amongst contaminants, which could be easily subjected to subculture for further identification). Some examples of agar plates representing the different classifications are shown in the Supplementary Data, Fig. 1.

Rapid detection protocol:The collected nBAL samples were vortexed for 30 seconds and 5 mL of each sample was transferred to a 15 mL falcon tube. After centrifugation (5152 × g for 10 minutes), the supernatant was carefully aspirated, leaving +/− 1 mL of cell pellet in the falcon tube. The cell pellet was vortexed and placed in a 50 mL falcon tube containing 10 mL BHIB supplemented with 10% FBS, 0.5% YE and 32 µg/mL bacitracin. All tubes were placed in a shaking incubator (35 °C +/−2 °C, 5% CO_2_) for 6 hours. After incubation, samples were centrifuged at 5152 × g for 10 minutes and the supernatant was aspirated, again leaving +/−1 mL of cell pellet in the falcon tube. For each sample, protein extraction and MALDI-TOF MS analysis was performed as previously described^15^. Briefly, the cell pellet in the falcon tube was transferred to an eppendorf tube and was centrifuged at 21130 × g for 5 minutes at room temperature. After centrifugation, the supernatant was carefully aspirated and 700 µL of 70% ethanol in high performance liquid chromatography (HPLC) graded water was added to the cell pellet and vortexed. A second centrifugation step with aspiration of the supernatant was performed as described above. After air drying for approximately 10 minutes, 20 µL 70% formic acid (in HPLC graded water) was added to the cell pellet and mixed carefully. Samples were incubated for five minutes at room temperature. In a last step, 20 µL of acetonitrile was added and vortexed. A third centrifugation step (21130 × g for 2 minutes) was performed to clarify the lysates. Noteworthy, the volume of formic acid and acetonitrile was adjusted to the size of the cell pellet (10 µL, 20 µL or 30 µL for a small, medium or large pellet, respectively, according to manufacturer’s guidelines).

One µL of the protein extraction was spotted in duplicate on a target plate (MSP 96 target polished steel BC). After air drying, one µL of matrix (10 mg/mL of alpha-cyano-4-hydroxy-cinnamic acid (alpha-HCCA) in 50% acetonitrile −47.5% water −2.5% trifluoroacetic acid; Bruker Daltonik GmbH, Bremen, Germany) was placed on each spot. External calibration was included using a bacterial test standard (BTS, Bruker Daltonik GmbH, Bremen, Germany). Analysis was performed with an Autoflex speed MALDI-TOF/TOF MS instrument (Bruker Daltonik GmbH, Bremen, Germany) with commercial software (flexControl 1.4, version 3.4., Bruker Daltonik GmbH, Bremen, Germany), recording the mass range between 2,000–20,000 Dalton using standard settings. The spectra were analysed using MBT Compass version 4.1 (Bruker Daltonik GmbH, Bremen, Germany) that included a reference database of 7926 different bacterial entries, using standard settings. Log score values < 1.7 represent no organism identification possible, a (log) score value between 1.7 and 2.0 represents identification at species level at low confidence, and a (log) score value ≥2.0 represents identification at species level at high confidence. The threshold for correct identification was determined at a (log) score value of ≥1.7, as previously described for rapid detection protocols in positive blood cultures^25,26^.

Diagnostic accuracy (sensitivity (Se) and specificity (Sp)) was determined with bacterial culture as reference test (Winepiscope 2.0 (Zaragoza, Spain)). Pure, dominant and mixed culture results were considered a positive outcome. Negative and polymicrobial test results were considered negative outcomes. Correct identification for 1 pathogen by MALDI-TOF MS in mixed cultures was considered a positive outcome. ‘No peaks found’, ‘no organism identification possible’ or identification of a contaminant by MALDI-TOF MS for negative and polymicrobial test results was considered a positive outcome.

## Supplementary information

Supplementary data, Table 1 and Figure 1

## Data Availability

The authors declare that all information obtained from this study is presented in this paper.
