# Supplementary material for: Rapid identification of respiratory bacterial pathogens from bronchoalveolar lavage fluid in cattle by MALDI-TOF MS
Source: Sci Rep. 2019 Dec 5;9:18381. doi: 10.1038/s41598-019-54599-9 (PMC6895124; doi:10.1038/s41598-019-54599-9)

Rapid identification of respiratory bacterial pathogens from bronchoalveolar lavage fluid in cattle by MALDI-TOF MS

Laura Van Driessche[1]*, Jade Bokma[1], Piet Deprez[1], Freddy Haesebrouck[2], Filip Boyen[2$], Bart Pardon[1$]

Table 1: Overview of the identification results of the conventional cultivation method on agar plate and the rapid MALDI-TOF MS method obtained during the validation study

Sample number	Classification conventional culture	Pathogens (concentration, CFU/mL)	Contaminants (concentration, CFU/mL)	Rapid MALDI-TOF MS identification	(Log) score value
**1**	Mixed: *M. haemolytica, B. trehalosi*	*M. haemolytica*: 5x10^3, *B. trehalosi*: 4x10^2	*Aerom.veronii*: 2x10^1, *Bacillus spp.*: 2x10^1, *Staph. sciuri*: 2x10^1	*M. haemolytica*	2.49
**2**	Dominant: *M. haemolytica*	*M. haemolytica*: 5x10^2	*Staph. cohnii*: 2x10^2, *Bacillus spp.*: 2x10^1, *Coryn. amycolatum*: 6x10^1, *Strept. suis:* 2x10^2	*M. haemolytica*	2.32
**3**	Mixed: *M. haemolytica, M. ovis*	*M. haemolytica*: 6x10^4, *M. ovis*: 3x10^3	/	*M. haemolytica*	2.35
**4**	Dominant: *M. haemolytica*	*M. haemolytica*: 2x10^1	*Staph. sciuri*: 8x10^1, *Staph. lentus*: 2x10^2, *Neisseria perflava*: 4x10^2	*M. haemolytica*	2.40
**5**	Negative	/	/	No organism identification possible	1.47
**6**	Dominant: *M. haemolytica*	*M. haemolytica*: 4x10^2	*Bacillus spp.*: 1x10^3	*M. haemolytica*	2.03
**7**	Pure: *M. haemolytica*	*M. haemolytica*: 2x10^3	/	*M. haemolytica*	2.39
**8**	Polymicrobial	/	*Staph. rostri*: 1x10^3, *E. coli*: 2x10^3	*E. coli*	2.26
**9**	Mixed: *B. trehalosi, M. haemolytica*	*B. trehalosi*: 4x10^3, *M. haemolytica*: 1x10^2	*Strept. hyovaginalis*: 1x10^2	*B. trehalosi*	2.29
**10**	Mixed: *M. haemolytica, B. trehalosi, M. varigena, H. somni*	*M. haemolytica*: 1x10^3, *B. trehalosi*: 1x10^3, *H. somni*: 5x 10^4, *M. varigena*: 7x10^3	*E. coli*: 1x10^2	*B. trehalosi*	1.91
**11**	Mixed: *M. haemolytica, H. somni, M. ovis*	*M. haemolytica*: 2x10^3, *H. somni*: 1x10^4, *M. ovis*: 3x10^4	/	No organism identification possible	1.23

**12**	Mixed: *M. haemolytica*, *B. trehalosi*	*M. haemolytica*: $5x10^4$, *B. trehalosi*: $2x10^4$	*Bacillus spp.*: $2x10^3$	*M. haemolytica*	2.15
**13**	Pure: *B. trehalosi*	*B. trehalosi*: $1x10^6$	/	*B. trehalosi*	2.05
**14**	Pure: *H. somni*	*H. somni*: $4x10^4$	/	No organism identification possible	1.69
**15**	Pure: *H. somni*	*H. somni*: $1x10^3$	/	No organism identification possible	1.23
**16**	Mixed: *B. trehalosi*, *H. somni*, *M. haemolytica*	*B. trehalosi*: $5x10^2$, *H. somni*: $2x10^3$, *M. haemolytica*: $1x10^2$	/	*B. trehalosi*	2.12
**17**	Mixed: *H. somni*, *M. haemolytica*	*H. somni*: $1x10^3$, *M. haemolytica:* $3x10^1$	*Staph. equorum*: $2x10^1$	No organism identification possible	1.44
**18**	Mixed: *H. somni*, *M. ovis*, *M. varigena*	*H. somni*: $4x10^4$, *M. varigena*: $8x10^3$, *M.ovis*: $1x10^3$	/	*H. somni*	1.71
**19**	Mixed: *M. haemolytica*, *P. multocida*	*M. haemolytica*: $4x10^4$, *P. multocida*: $3x10^3$	/	*M. haemolytica*	2.14
**20**	Mixed: *M. haemolytica*, *B. trehalosi*, *M. varigena*	*M. haemolytica*: $1x10^2$, *B. trehalosi*: $2x10^2$, *M. varigena*: $5x10^1$	*Strept. hyovaginalis*: $1x10^3$, *Neisseria subflava*: $2x10^3$, *Staph. xylosus*: $3x10^2$	*B. trehalosi*	2.24
**21**	Dominant: *G. anatis*	*G. anatis*: $7x10^3$	*Bacillus spp.*: $6x10^2$, *Lactobacillus salivarius*: $2x10^3$, *Pelistega europaea*: $2x10^4$	*G. anatis*	2.23
**22**	Mixed: *M. haemolytica*, *H. somni*	*M. haemolytica*: $8x10^2$, *H. somni*: $3x10^2$	*Staph. equorum*: $8x10^2$	*Staph. equorum*	1.73
**23**	Dominant: *M. haemolytica*	*M. haemolytica*: $6x10^4$	*Staph. aureus*: $1x10^2$	*M. haemolytica*	2.21
**24**	Negative	/	/	No organism identification possible	1.59
**25**	Negative	/	/	No peaks found	0
**26**	Polymicrobial	/	*Strept. suis*: $1x10^3$, *Bacillus spp.:* $1x10^3$	No organism identification possible	1.5
**27**	Dominant: *M. ovis*	*M. ovis*: $2x10^2$	*Staph. haemolyticus*: $1x10^1$	No organism identification possible	1.46

**28**	Negative	/	*Staph. haemolyticus*: $1x10^1$	No organism identification possible	1.69
**29**	Negative	/	/	*Staph. haemolyticus*	1.88
**30**	Dominant: *M. ovis*	*M. ovis*: $2x10^3$	*Staph. chromogenes*: $1x10^2$	No organism identification possible	1.56
**31**	Polymicrobial	/	*Staph. xylosus*: $4x10^1$, *Neisseria flavescens*: $2x10^2$, *Strept. suis*: $4x10^2$	No organism identification possible	1.68
**32**	Mixed: *M. ovis*, *P. multocida*	*M. ovis*: $4x10^4$, *P. multocida*: $1x10^3$	/	*Staph. lentis*	1.77
**33**	Mixed : *M. ovis*, *P. multocida*	*P. multocida*: $9x10^3$, *M. ovis*: $1x10^3$	/	*Staph. haemolyticus*	1.74
**34**	Dominant: *M. varigena*	*M. varigena*: $3x10^1$	*E. coli*: $1x10^2$, *Neisseria subflava*: $1x10^3$, *Strept. suis*: $3x10^3$	No organism identification possible	1.62
**35**	Polymicrobial	/	*Acinetobacter towneri*: $3x10^1$, *Serratia liquefaciens*: $4x10^2$, *Aerococcus viridans*: $9x10^1$	No organism identification possible	1.61
**36**	Dominant: *M. ovis*	*M. ovis*: $3x10^2$	*Staph. sciuri*: $8x10^1$	No organism identification possible	1.26
**37**	Mixed: *P. multocida*, *M. haemolytica*, *M. ovis*	*P. multocida*: $2x10^3$, *M. ovis*: $4x10^3$, *M. haemolytica:* $5x10^3$	*Strept. suis*: $3x10^2$	No organism identification possible	1.56
**38**	Pure: *P.multocida*	*P. multocida*: $7x10^4$	/	*P. multocida*	2.01
**39**	Dominant: *G. anatis*	*G. anatis*: $1x10^4$	*Strept. suis*: $5x10^2$	*G. anatis*	2.28
**40**	Pure: *Staph. sciuri*	/	*Staph. sciuri*: $6x10^1$	*Staph. equorum*	1.73
**41**	Polymicrobial	/	*Morganella morganii*: $5x10^1$, *Lysinibacillus fusiformis*: $1x10^1$	No peaks found	0
**42**	Pure: *H. somni*	*H. somni*: $5x10^2$	/	No organism identification possible	1.5
**43**	Pure: *M. haemolytica*	*M. haemolytica*: $1x10^4$	/	*M. haemolytica*	2.33
**44**	Dominant: *P. multocida*	*P. multocida*: $2x10^3$	*Bacillus spp*: $2x10^3$, *Strept. dysgalactiae*: $2x10^2$	*P. multocida*	2.37

**45**	Polymicrobial	/	*Pantoea agglomerans*: $1x10^1$, *Staph. fleurettii*: $1x10^3$, *Bacillus spp*: $1x10^1$	No peaks found	0
**46**	Negative	/	/	No organism identification possible	1.6
**47**	Mixed: *H. somni*, *M. haemolytica*	*H. somni*: $1x10^4$, *M. haemolytica*: $2x10^3$	*Strept. suis*: $7x10^2$	*Strept. suis*	2.23
**48**	Negative	/	/	No peaks found	0
**49**	Dominant: *M. haemolytica*	*M. haemolytica*: $1x10^4$	*E. coli*: $2x10^4$, *Strept. suis*: $4x10^3$	*E. coli*	2.33
**50**	Negative	/	*E. coli*: $1x10^1$	No peaks found	0
**51**	Polymicrobial	/	*Lysinibacillus fusiformis*: $5x10^1$, *Providencia rettgeri*: $5x10^2$	*E. coli*	2.18
**52**	Dominant: *M. haemolytica*	*M. haemolytica*: $1x10^5$	*Strept. suis*: 1x10^4	*M. haemolytica*	2.09
**53**	Dominant: *B. trehalosi*	*B. trehalosi*: $3x10^1$	*Bacillus spp*: $1x10^1$	*B. trehalosi*	1.99
**54**	Negative	/	/	No peaks found	0
**55**	Polymicrobial	/	*Bacillus spp*: $2x10^3$, *Strept. pluranimalium*: $5x10^1$, *E. coli*: $4x10^1$	No organism identification possible	1.27
**56**	Polymicrobial	/	*Bacillus spp*: $6x10^1$, *Strept. pluranimalium* $3x10^2$, *Staph. chromogenes*: $1x10^1$	No organism identification possible	1.46
**57**	Dominant: *M. varigena*	*M. varigena*: $2x10^3$	*Bacillus spp.*: $3x10^1$, *Strept. pluranimalium*: $7x10^1$	No organism identification possible	1.31
**58**	Negative	/	/	No organism identification possible	1.38
**59**	Mixed : *P. multocida*, *M. ovis*	*P. multocida*: 10^6, *M. ovis*: $4x10^3$	*Strept. suis*: $7x10^2$	*P. multocida*	2.02
**60**	Mixed: *M. ovis*, *P. multocida*	*M. ovis*: $4x10^3$, *P. multocida*: $8x10^2$	*Strept. pluranimalium*: $3x10^2$	No organism identification possible	1.44
**61**	Dominant: *M. ovis*	*M. ovis*: $6x10^2$	*Neisseria meningitidis*: $5x10^2$	No organism identification possible	1.17
**62**	Dominant: *M. ovis*	*M. ovis*: $6x10^3$	*Strept. suis*: $2x10^1$	No organism identification possible	1.61
**63**	Negative	/	/	No organism identification possible	1.17

**64**	Mixed : *M. ovis, P. multocida*	*P. multocida*: $1x10^8$, *M. ovis*: $4x10^2$	/	*P. multocida*	1.71
**65**	Dominant: *M. ovis*	*M. ovis*: $1x10^4$	*Strept. pluranimalium*: $1x10^2$	No organism identification possible	1.2
**66**	Negative	/	*Acinetobacter lwoffii*: $1x10^1$	No organism identification possible	1.25
**67**	Pure: *M. ovis*	*M. ovis*: $4x10^3$	/	No organism identification possible	1.23
**68**	Negative	/	*Strept. equorum*: $1x10^1$	No organism identification possible	1.48
**69**	Pure: *P. multocida*	*P. multocida*: $1x10^4$	/	*P. multocida*	2.24
**70**	Negative	/	/	*Staph. lentus*	1.73
**71**	Dominant: *B. trehalosi*	*B. trehalosi*: $4x10^2$	*Staph. equorum*: $3x10^1$, *Neisseria flavescens*: $8x10^1$, *Strept. suis*:$2x10^1$	*B. trehalosi*	1.88
**72**	Polymicrobial	/	*Pantoea anatis*: $2x10^2$, *Bacillus spp.:* $1x10^1$, *Strept. suis* $2x10^1$, *Staph. chromogenes*: $3x10^1$	No organism identification possible	1.65
**73**	Dominant: *P. multocida*	*P. multocida*: $3x10^3$	*Staph. chromogenes*: $1x10^3$	*P. multocida*	1.78
**74**	Mixed: *M. haemolytica, M. ovis*	*M. haemolytica*: $4x10^2$, *M. ovis*: $2x10^2$	*Strept. suis*: $1x10^1$	No organism identification possible	1.42
**75**	Dominant: *P. multocida*	*P. multocida*: $3x10^3$	*Staph. chromogenes*: $3x10^2$, *Strept. pluranimalium*: $1x10^2$	*P. multocida*	1.72
**76**	Dominant: *P. multocida*	*P. multocida*: $2x10^3$	*Staph. chromogenes*: $1x10^3$, *Strept. suis*: $1x10^3$, *Neisseria flavescens*: $5x10^2$	*P. multocida*	1.74
**77**	Pure: *M. varigena*	*M. varigena*: $5x10^1$	/	*M. varigena*	2.00
**78**	Mixed: *M. haemolytica, M. ovis*	*M. haemolytica*: $7x10^1$, *M. ovis*: $3x10^2$	*Strept. suis*: $1x10^2$	*M. haemolytica*	2.02
**79**	Mixed: *P. multocida, M. ovis*	*P. multocida*: $1x10^4$, *M. ovis*: $8x10^3$	*Staph. xylosus*: $1x10^2$, *Strept. suis*: $1x10^3$	*P. multocida*	2.33
**80**	Pure: *E. coli*	*E. coli*: $4x10^2$	/	*E. coli*	2.13
**81**	Mixed: *M. ovis, M. varigena*	*M. ovis*: $4x10^3$, *M. varigena*: $4x10^3$	*Staph. chromogenes*: $3x10^3$	No organism identification possible	1.35

**82**	Mixed: *M. haemolytica, P. multocida*	*M. haemolytica*: 3x10^{1}, *P. multocida*: 8x10^{1}	*Bacillus spp.*: 1x10^{1}, *Strept. suis*: 2x10^{1}, *Staph. Aureus* : 6x10^{1}	*M. haemolytica*	1.74
**83**	Polymicrobial	/	*Strept. suis*: 2x10^{1}, *E. coli*: 6x10^{1}	No organism identification possible	1.30
**84**	Mixed: *M. haemolytica, P. multocida*	*M. haemolytica*: 3x10^{1}, *P. multocida*: 4x10^{1}	*Neisseria spp.*: 2x10^{2}, *Strept. suis*: 2x10^{1}	*M. haemolytica*	2.03
**85**	Negative	/	/	No organism identification possible	1.34
**86**	Polymicrobial	/	*E. coli*: 1x10^{2}, *Bacillus spp.*: 1x10^{1}, *Staph. xylosus* : 8x10^{1}, *Strept. suis* : 1x10^{2}	*E. coli*	2.08
**87**	Polymicrobial	/	*E. coli*: 3x10^{2}, *Bacillus spp.*: 2x10^{1}, *Staph. haemolyticus*: 1x10^{2},	*E. coli*	2.23
**88**	Polymicrobial	/	*Staph. chromogenes*: 7x10^{2}, *Bacillus spp.*: 1x10^{1}, *Strept. suis*: 1x10^{2}, *Kluyvera intermedia*: 1x10^{2}	No organism identification possible	1.35
**89**	Dominant: *M. haemolytica*	*M. haemolytica*: 3x10^{2}	*E. coli*: 2x10^{3}	No organism identification possible	1.3
**90**	Mixed: *P. multocida, M. haemolytica*	*P. multocida*: 1x10^{4}, *M. haemolytica*: 6x10^{2}	*Strept. suis*: 4x10^{3}	*P. multocida*	2.17
**91**	Polymicrobial	/	*Strept. pluranimalium*: 2x10^{1}, *Staph. aureus*: 8x10^{1}	No organism identification possible	1.45
**92**	Pure: *P. multocida*	*P. multocida*: 2x10^{4}	/	*P. multocida*	2.32
**93**	Polymicrobial	/	*Lactobacillus spp.*: 4x10^{2}, *Staph. xylosus*: 9x10^{1}	No organism identification possible	1.40
**94**	Polymicrobial	/	*E. coli*: 6x10^{1}, *Strept. suis*: 1x10^{2}, *Neisseria spp.*: 2x10^{2}	No organism identification possible	1.29
**95**	Polymicrobial	/	*Lysinibacillus fusiformis*: 1x10^{2}, *Staph. chromogenes*: 2x10^{2}	No organism identification possible	1.52
**96**	Mixed: *M. varigena, H. somni*	*M. varigena*: 8x10^{1}, *H. somni*: 1x10^{2}	/	*Staph. haemolyticus*	1.7

**97**	Mixed: *M. ovis*, *H. somni*	*M. ovis*: 7x10^2, *H. somni*: 1x10^3	/	*E. coli*	1.81
**98**	Negative	/	/	no peaks found	0
**99**	Dominant: *M. ovis*	*M. ovis*: 3x10^4	*E. coli*: 2x10^2	*E. coli*	1.77
**100**	Pure: *Pseudomonas aeruginosa*	/	*Pseudomonas aeruginosa*: 5x10^1	*Pseudomonas aeruginosa*	1.92

Figure 1: visualisation of the classification of clinical nBAL samples from cattle obtained by conventional culture

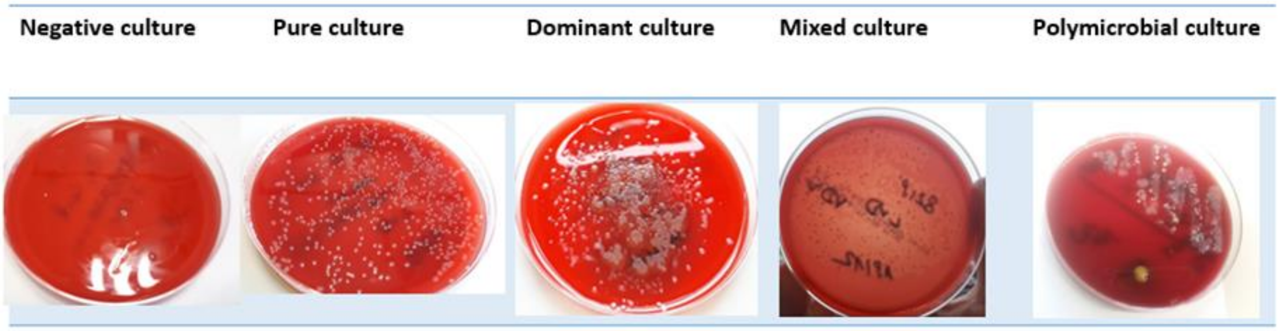

Supplement: Supplementary file 1 — Supplementary data, Table 1 and Figure 1 [file 41598_2019_54599_MOESM1_ESM.pdf]
